# “Is the Service Ready?”: Integrating Peer Support Workers Within Community Mental Health: An Ethnographic Study from Trieste and its Region

**DOI:** 10.1007/s10597-025-01513-5

**Published:** 2025-09-16

**Authors:** Giulia Pollice, Chiara Francesca Bodini, Marco Menchetti, Delia Da Mosto, Luca Negrogno, Lorenzo Betti, Morena Furlan, Ivo Quaranta

**Affiliations:** 1 University of Modena and Reggio Emilia, Modena, Italy; 2 Centro di Salute Internazionale e Interculturale - APS, Bologna, Italy; 3 University of Bologna, Bologna, Italy; 4 Istituzione Gian Franco Minguzzi, Città Metropolitana di Bologna, Bologna, Italy; 5 Central Health Directorate, Friuli Venezia Giulia Region, Trieste, Italy

**Keywords:** Peer support workers, People with lived experience, Community psychiatry, Recovery-oriented practices, Mental health services, Ethnography.

## Abstract

Peer support, endorsed by WHO and national guidelines, is increasingly recognised as a key component of mental health care innovation. However, while peer support has gained increasing attention, the effective integration of Peer Support Workers (PSWs) within mental health services—and the systemic challenges it involves—remains a relatively under-investigated area. This study investigates the integration of PSWs in Trieste and its region, a pivotal site of Italy’s psychiatric reform, where the deinstitutionalisation movement fostered the transition to a community-based care model. The present study adopted an ethnographic methodology, encompassing a six-month field study involving participant observation and 22 semi-structured interviews with 12 PSWs and 10 mental health professionals. PSWs contribute to enhancing empathy, user engagement, and social inclusion, while also fostering a recovery and community-oriented approach to care. However, challenges have emerged, including role ambiguity, institutional under-recognition, and professional resistance. These tensions often reflect broader issues around power dynamics and the epistemic legitimacy of lived experience. The study also identifies strategies to support PSW integration, including safeguarding practices, interprofessional training, and institutional recognition. In addition to their clinical and relational contributions, PSWs offer an opportunity for mental health services to critically reflect on their practices, assumptions, and power structures. Their meaningful inclusion can catalyse a shift towards more participatory, rights-based, and recovery-oriented care.

## Introduction

The World Health Organisation, the Lancet Commission on Global Mental Health and various national and international guidelines emphasise the necessity of reforming mental health services by prioritising care that values lived experience and safeguards individuals’ rights (World Health Organisation, 2019; Rose, 2014; Napier et al., 2014; Patel et al., 2018). A pivotal player in this process is the Peer Support Worker (PSW); PSWs are individuals with lived experience of mental disorders who help others currently facing similar difficulties (Repper & Carter, 2011a). The role of PSWs encompasses a wide spectrum of activities, such as providing individual support, facilitating peer support groups or shadowing professionals (Lloyd-Evans et al., 2014; Repper, 2013; Repper & Carter, 2011a). The efficacy of peer support has thus far demonstrated favourable outcomes concerning clinical (Cooper et al., 2024; Høgh Egmose et al., 2023) and personal recovery, fostering empowerment, self-efficacy, feelings of hope, self-esteem and social integration (Burke et al., 2019; Chien et al., 2019; Cooper et al., 2024). The growing relevance of PSWs in mental health systems is reflected in their consistent inclusion within international guidelines and policy recommendations (Bologna & Simmons, 2018; National Institute for Health and Care Excellence (NICE), 2014; Ostrow & Adams, 2012; World Health Organisation, 2019). In several countries—such as the United Kingdom, the United States, Canada, New Zealand and Australia—PSWs have been formally recognised as key actors within recovery-oriented systems of care (Bologna & Simmons, 2018; Foglesong et al., 2022; Kaufman et al., 2014; Mental Health Commission, 2012; Ostrow & Adams, 2012; UK Department of Health, 2011). Their integration is supported by national strategies that include formal certification processes, targeted training programmes, and employment frameworks. These developments align with a broader paradigm shift toward participatory and rights-based models in mental health care, which recognise the epistemic legitimacy of lived experience as a necessary complement to professional and clinical knowledge.

Nonetheless, several important aspects of PSW implementation remain insufficiently understood. Many studies still offer only vague or inconsistent descriptions of PSWs’ roles, with limited attention given to whether their activities align with the core values and distinctive skills of peer support work (Bologna & Simmons, 2018; Cooper et al., 2024; Gillard et al., 2017, 2024). Moreover, there is a notable gap in research examining how outcomes associated with peer support relate to the actual conditions under which PSWs operate—such as the organisational structure of services and the readiness of professionals to collaborate effectively (Cooper et al., 2024; Gillard et al., 2024). While PSWs are often framed as agents of transformation, it remains unclear whether their integration truly drives cultural and structural change or whether it instead mirrors a broader but superficial shift in professional rhetoric and practice (Bologna & Simmons, 2018; Gillard et al., 2017, 2024). These gaps highlight the growing need for qualitative, context-sensitive research to explore both the enabling factors and the obstacles that shape the meaningful implementation of peer support (Høgh Egmose et al., 2023).

### Italian Context

In Italy, the role of the PSW (translated into Italian as *Persona Esperta in Supporto tra Pari*, abbreviated as ESP) has developed in diverse forms over the past two decades, through a dynamic and often unstructured process of experimentation. Initiatives involving the participation of individuals with lived experience in peer support activities have emerged independently across various local contexts, shaped by the cultural substrate of each territory.

In 2020, the "National Network of PSWs" was established, initiating a collaborative process involving 43 organisations across 11 Italian regions. This effort led to the formal definition of the PSW role through the development of the "Italian National Charter of PSWs" (AIPESP, 2021), and subsequently, in 2024, to the founding of the "Italian Association of PSWs"of (*Associazione Italiana Persone Esperte in Supporto tra Pari*,* AIPESP*)^1^. The professional profile and core values endorsed by the Association align with the principles of peer support outlined in the World Health Organisation’s QualityRights programme (2019).

According to a national survey conducted by the Italian Association of PSWs, there is considerable heterogeneity in both the activities undertaken by PSWs and their employment arrangements. The most frequently reported activities include sharing personal testimony and raising awareness, providing initial reception and orientation, and facilitating group workshops. The majority of PSWs are engaged through third-sector organisations such as associations and social cooperatives, rather than being directly employed by the public health system (Guzzetta et al., 2024).

These findings underscore the need for a deeper understanding of how the PSW role operates within specific local contexts—its boundaries, challenges, and potential impact on the effectiveness and efficiency of mental health services.

### Research Context – Friuli Venezia Giulia Region

The study was carried out in the community mental health services of the Friuli Venezia Giulia region in Italy, which was the pivotal site in the genesis of the Italian deinstitutionalisation process. This movement had national and international reach and led to a profound transformation in psychiatric healthcare through the abolition of asylums and the development of a rich network of community care focused on promoting users' empowerment and self-determination (Muusse et al., 2020; Portacolone et al., 2015; Sashidharan, 2022). To this day, the city of Trieste, the region’s main urban centre, continues to function as a vanguard, as evidenced by its WHO collaborating centre for mental health research (Mezzina, 2016) and an enhanced community care network (Bono et al., 2024). The services in Trieste are defined by a collaborative, multidisciplinary approach involving mental health professionals and patients in designing and delivering care (Bono et al., 2024). Furthermore, Trieste’s mental health system emphasises the importance of social determinants of health and focuses on integrating mental health services with other social services, providing a multidimensional and person-centred approach to care (Mezzina, 2014, 2016). The core of the organisation is a network of "*Community Mental Health Centres*" that are active 24 hours a day, 7 days a week, with relatively few beds in each of them; this organisation is characterised by an “open door” and “no restraint” approach (Mezzina, 2014). The regional government of Friuli Venezia Giulia replicated Trieste’s model across the region, resulting in low hospitalisation and compulsory treatment rates, effective job placement and social inclusion (Mezzina, 2016).

Consequently, Trieste and its region provide an ideal context for investigating the role of PSWs, analysing the factors driving their involvement, the strategies that have been deployed, the critical issues that have emerged in comparison with other contexts, and whether valuable insights can be drawn from this dynamic landscape of innovation.

To explore these issues while remaining attentive to the structural dynamics affecting PSWs and professionals, we adopted an ethnographic approach that allows for an in-depth exploration of the lived experiences of PSWs and professionals (Savage, 2000, 2006).

### Aims

1. Understand the integration of PSWs through an in-depth analysis of their current experience in the Friuli Venezia Giulia region.
2. Identify the strengths and challenges of the inclusion of PSWs in mental health services.
3. Outline future perspectives and pathways to ensure that PSWs’ engagement is effective for service users and enriching for the service, fostering a recovery-oriented approach.

## Methods

### Study Design

We conducted the following ethnographic study: (1) Participant observation: the principal researcher undertook a six-month observation period, attending meetings and events organised by the “PSWs Regional Network”, participating in an action-research training course for professionals, family members and PSWs, and shadowing two PSWs in Trieste. (2) Concurrently, the principal researcher conducted 22 semi-structured interviews with PSWs and mental health professionals (MHPs). Each interview lasted approximately 60 min and was carried out in Italian. The findings were periodically reviewed and discussed by the research team. The subjects addressed during the interviews encompassed the following: the role of PSWs within the service, the perceived positive outcomes, the critical issues encountered, and the resilience strategies employed or deemed appropriate for implementation. The interviews were conducted in April and May 2024. Informed consent for anonymous data processing was obtained before the interviews were recorded.

### Recruitment and Participants

The recruitment process began during the participant observation phase, when the first PSWs were identified and interviewed. All subsequent participants were recruited via the snowball sampling method (Parker et al., 2019). Following grounded theory principles (Glaser & Strauss, 2017), all PSWs active in Friuli Venezia Giulia during the data collection were included. For MHPs, the inclusion criterion was having direct experience of working with PSWs. The number of interviews with MHPs was defined by the principle of theoretical saturation, which is consistent with grounded theory methodology.

### Data Analysis

All interviews were transcribed verbatim and anonymised, with participants identified as either MHPs or PSWs to provide contextual clarity in the presentation of quotes. Data analysis followed a deductive–inductive logic and was conducted using a framework analysis approach (Ritchie & Spencer, 1994) , which allowed for both structured comparison across participant groups and openness to themes emerging from the data.

The process was conducted manually, to ensure deep and sustained engagement with the material and to foster a nuanced understanding of participant narratives that might be less apparent with the use of automated software. As emphasised in the literature, manual approaches can offer several advantages in such contexts, including reduced complexity, greater interpretative depth, and increased proximity to the data, particularly in small-scale qualitative studies such as ours, where the volume of material allows for close and iterative engagement (Basit, 2003). Moreover, manual coding has been shown to facilitate a more flexible and responsive analytic process, supporting the identification of subtle thematic patterns and enhancing the interpretive fidelity of the analysis (Basit, 2003). This approach also aligned with the collaborative and dialogical nature of the research team’s analytic process, enabling continuous refinement of the coding matrix.

Based on the study’s research aims and an initial close reading of the transcripts, a preliminary coding matrix was developed by the first author, encompassing macro-themes and sub-themes that reflected recurring issues across the narratives of both PSWs and MHPs. This matrix was subsequently reviewed and refined in collaboration with two additional members of the research team. As the analysis progressed, new sub-themes were inductively identified and incorporated into the evolving matrix.

Coding decisions and thematic interpretations were discussed iteratively among the researchers to ensure analytic coherence, and a shared interpretative framework was developed through consensus. The data were systematically charted within this framework, allowing for both in-depth analysis within each participant group and comparative examination across groups, with particular attention to points of convergence and divergence.

At the conclusion of the analytical process, a written summary report of the findings was shared with participants. Their feedback was explicitly solicited and used to inform a final revision of both the coding matrix and the interpretation of results. 17 of the 22 participants—including 9 of the 12 PSWs—provided comments on the findings. This member validation phase served as an additional layer of analytical refinement and contributed to enhancing the credibility, contextual relevance, and trustworthiness of the study.

### Research Team

The research team was interdisciplinary, comprising four members with primarily biomedical backgrounds and three members with socio-anthropological expertise. Specifically, the team included: one medical doctor with academic training in medical anthropology and ethnography; one anthropologist specialised in medical anthropology; one public health specialist with expertise in qualitative research in mental health; one psychiatrist; one medical doctor and anthropologist; one sociologist specialised in participatory research and user involvement in mental health services; one sociologist with expertise in health promotion and the Trieste mental health system; and one psychiatric rehabilitation technician with managerial responsibilities at the regional level in Friuli Venezia Giulia. This diverse composition enabled a plurality of perspectives and ensured that both clinical and socio-cultural dimensions of peer support were critically addressed. Although none of the researchers identified as PSW or persons with lived experience, findings and interpretations were shared and discussed with participants throughout the analysis phase to enhance credibility and ensure contextual relevance. Throughout the research process, particular attention was paid to power asymmetries within the team and in relation to participants. Regular reflexive discussions were held to critically examine positionalities and interpretive assumptions. Furthermore, the participatory structure of the analysis—especially the incorporation of feedback from PSWs and MHPs—was intended to mitigate hierarchical dynamics and promote shared meaning-making.

## Results

### Participants

Table 1 summarises the professional profile of the PSWs and MHPs interviewed.

**Table 1:** Participants

12 PSWs	4 PSWs employed on a contract basis in two social cooperatives. 3 PSWs volunteering in associations. 5 PSWs on a traineeship in mental health services.
10 MHPs	3 psychiatrists. 1 psychologist. 2 psychiatric rehabilitation specialists. 1 social worker. 1 public health physician. 1 nurse. 1 professional educator.

### Analysis of Interviews

To facilitate understanding, the results collected were grouped and classified by macro themes, as shown in Table 2.

**Table 2:** Results overview

Activities	Group activities for service-users. Advocacy and community engagement. Training for MHPs. Sensibilisation of general society. Recovery House. One-to-one meetings.
Benefits	Foster empathy, understanding and reduction of self-stigma. Promote social inclusion by assisting in daily life activities and facilitating the creation of social networks. Enhance the person’s point of view with professionals. Advocacy for the protection of rights. Foster PSW’s own recovery.
Challenges	Stigma and prejudice from MHPs. Lack of formal recognition and misuse of PSWs. Power imbalances and resistance to change from MHPs. Instrumentalization and manipulation of PSWs.
Strategies for inclusion	Ensure a safeguarding work environment. Training for MHPs to work with PSWs. Institutional recognition of PSWs’ role. Work in recovery-oriented settings. Synergy between operational motivation and strategic institutional support. Cultural and epistemological change in mental health services.

#### Activity Profile

PSWs were asked to describe their activities. The results are summarised in Table 3.

**Table 3:** Activity profile

Group activities	Weekly or biweekly peer support groups. Peer support in Trieste’s psychiatric ward for acute cases. Creative workshops (writing, art, cinema). Group activities for social integration (excursions, dinners, out-of-town trips of one or more days). Weekly meetings in the acute hospital ward.
Advocacy and Community engagement	Peer support groups in the suburbs of Trieste. Citizenship awareness activities (conferences and meetings open to the public).
Teaching	Academic courses at the University of Trieste. Training for MHPs.
Collaboration with MHPs	Weekly reports and monthly meetings with some MHPs: in which the needs identified by the PSWs in the groups they facilitate during the week are reported periodically, as well as cases and events that need special attention from the service.
Recovery House	Support for daily activities, presence, and active listening with the young residents of a Recovery House. Assistance in recovery programmes such as the *Wellness Recovery Action Plan.*
One-to-one meetings	Individual meetings upon request of the service users.

#### Benefits of PSWS

1. Fostering empathy, understanding, and reducing self-stigma

PSWs play a crucial role in actively listening to and understanding distress. They recognise the importance of being present, non-judgmental, and validating emotions. As one PSW noted:

*“PSWs know that it is often unhelpful to say ‘Do it! You can do it!’. Sometimes*,* what matters most is standing there in silence*,* without judgment*,* and conveying the message that ‘it is okay to feel unwell’”. (PSW5).*

Through their lived experience, PSWs foster hope and help reduce self-stigma, particularly for individuals in the early stages of recovery, who may feel lost and struggle to believe in their resources. The interviewees emphasised the role of PSWs in *normalising* mental health conditions, as well as their ability to carry out activities that cultivate peer relationships, ultimately encouraging active participation rather than passive service use.

2. Support for social inclusion

*“We do not do ‘for’ others but ‘with’ others”. (PSW8).*

According to the data, PSWs create opportunities for relationships founded on shared experiences, avoiding paternalistic and dependency-inducing support. Instead, they empower individuals to reclaim agency over their lives and their recovery, helping them move beyond the ‘patient’ identity. Furthermore, the study findings also suggest that resources for recovery are not only found within the individual but also enhanced through relationships and participation in group settings.

*“Participating in group meetings gave us an awareness of our worth. Sharing within the group makes you realise that you are worth it*,* despite the challenges your story matters”. (PSW5).*

3. Enhancing the person’s perspective with professionals

The interviewees highlighted the critical mediating role of PSWs in bridging communication between professionals and service users. The power imbalance created by professionals’ technical knowledge can sometimes hinder therapeutic effectiveness, as users may struggle to articulate their needs or concerns. For example, when individuals wish to adjust or suspend their medication, they often turn to PSWs, who facilitate discussions with psychiatrists. PSWs emphasised that their role is not about complicity but rather about supporting individuals in recognising and effectively communicating their needs to professionals.

MHPs reported that PSWs facilitate professionals in adopting a more human-centred approach, thereby fostering heightened sensitivity towards the individuals with whom they engage. Moreover, PSWs encourage practitioners to embrace risk-taking as part of the empowerment process of the individual, balancing it with necessary support. As one psychiatrist noted:

*“The PSW has a vision and a sensitivity that we sometimes lack. […] We worry about our responsibilities*,* we tend to intervene*,* to decide for people. I believe that the PSW experience helps me see things more from the patient’s perspective”. (MHP10).*

4. Advocacy for the protection of rights

The interviewed PSWs consistently emphasised the importance of advocacy in safeguarding service users’ rights. This was particularly evident during the participant observation period: in this region, PSWs’ advocacy is reflected both in their daily work within services, promoting a person-centred and rights-based approach, and in their involvement in the design and innovation of services and care practices. Moreover, many engage in activism and public awareness campaigns to inform communities about mental health services, reduce stigma, and promote a multidisciplinary understanding of mental illness.

5. Enhancing PSWs’ own recovery

Training and professional engagement in peer support constitute essential parts of PSWs’ personal recovery journeys. The process of recognising their own lived experiences as valuable tools for helping others reinforces their sense of empowerment. This transformation involves reframing suffering: rather than viewing mental illness as a stigmatising burden, PSWs reinterpret their experiences as a source of insight and resilience. As one participant shared:

*“That suffering is enormous*,* it is heavy and hurts*,* but when you make sense of things*,* it becomes your strength”. (PSW8).*

#### PSWs’ Challenges in Mental Health Services

1. Stigma and prejudice from MHPs

A key challenge identified in the study is the persistent stigma and prejudice harboured by some professionals towards PSWs. This is often expressed through overprotective or excessively cautious attitudes. One PSW described feeling like “*a problem to be solved*” (PSW4), as professionals continued to see him primarily as a former service user rather than acknowledging his skills and contributions. Several interviewees suggested that this prejudice stems from the ambiguity and perceived “threat” of PSWs’ role, which challenges the conventional *“us-healthy vs. them-sick dichotomy*” (PSW9).

2. Lack of formal recognition and misuse of PSW

In several interviews, an important theme emerged was that of the improper activities that PSWs could be called upon to perform. For example, we identified: (1) peer support as “employment inclusion”: the PSW is assigned menial tasks or duties unrelated to peer support; (2) PSWs as “low-cost” professional substitutes: sometimes, MHPs perceive PSWs as inexpensive replacements for trained professionals or as general-purpose workers who can perform tasks that mental health professionals prefer to avoid:

*“You have to be very careful because otherwise*,* they will offload tasks onto you that they should be doing themselves but don’t want to”. (PSW6).*

Moreover, in Italy, the role of PSWs lacks formal recognition, often resulting in internship-based employment with minimal guarantees. In Trieste, however, PSWs are employed through social cooperatives, offering greater security. Nonetheless, the interviewees emphasised the urgent need for greater institutional recognition.

3. Power imbalances and resistance to change

*“Is the service ready? Are we ready to question the services in which we work to involve them more?”. (MHP*,* March 2024*,* field diary).*

The interviewees highlighted that power imbalances between professionals and PSWs remain a major barrier to the full integration of PSWs. While PSWs have the potential to encourage professionals to critically reflect on their roles and practices, a reluctance to do so is often reported. One PSW described the difficulty of expressing a dissenting opinion within professional settings. Additionally, PSWs believe that this resistance stems from rigid role definitions and an unwillingness to move away from vertical and hierarchical dynamics, hindering the *therapeutic potential* of peer relationships.

*“Some professionals lack the humility to stop feeling superior to others — because it’s easier to feel legitimised by their professional role”. (PSW8).*

4. Instrumentalisation and manipulation of PSWs

Numerous interviewees stated the potential risk of instrumentalising PSW as a tool to “hook users” and “increase user compliance” with recovery programmes. Indeed, some PSWs perceive the risk of being manipulated as “*puppets in the hands of the system*” (PSW11). As one PSW described her experience during an internship at a suburban mental rehabilitation service that was inadequately prepared to involve PSWs:

*“They send me to talk to the users and afterwards I have to report what they tell me because the users speak a little bit more willingly with me than with the educator*,* so basically I am a pawn*,* they send me where they do not get”. (PSW10).*

Additionally, one psychiatrist criticised the potential risk of using PSWs instrumentally to give the service a more humane and avant-garde appearance. Consequently, it appears to be a “*concession”* (MHP4) of a space within which PSWs can act and express themselves:

*“The PSW should not be ‘in the service of the service’. However*,* often the current reality is like that*,* and it is difficult for it not to be so […] it is difficult for one to express criticism*,* suggestions*,* observations or to go into conflict because one would be ‘the last wheel on the wagon’ anyway*,* would not one?”. (PSW1).*

One MHP identified the risk that, despite the openness of services in contexts such as Trieste, the lack of clarity around the PSW role could enable subtle power imbalances and paternalistic attitudes to emerge, which may still limit the innovative potential of peer support by constraining PSWs within institutional structures.

*“In the beginning*,* the director wanted the PSWs and he was the one pushing it*,* […] but sometimes I have perceived it as: ‘Do it*,* but do it like this’ and ‘do it there*,* where it could also improve the image of the service’*,* […]. Therefore*,* perhaps in another context*,* it could have grown more. In my opinion*,* those who are ‘hungry’ are also more active […]; in other contexts where the ‘enemy’ is very clear*,* where the ‘enemy’ does not cooperate with you and does not tell you ‘Do this*,* do that…I’ll pay you’*,* maybe it is easier to create something real*,* heartfelt*,* with motivation”. (MHP7).*

#### Strategies for Effective Implementation

The study identified the following strategies as effective measures for safeguarding PSWs during their employment within mental health services and ensuring effective inclusion:

1. Ensuring safeguarding work inclusion

The respondents described several strategies implemented in Friuli Venezia Giulia as follows:

- Group and team work: Working in pairs or teams represents the main form of safeguarding, as it provides mutual *intervision*, helps identify moments of vulnerability and ensures support for the safety of both PSWs and users.
- Periodic supervision by a tutor or psychologist.
- Working in contexts separate from where one is/was a user helps PSWs establish professional relationships with mental health service colleagues while “*preserving the right to be ‘treated as a user’ in a different service”* (PSW12). This ensures adequate professional detachment, although it is not always practised, as seen in Trieste, where PSWs work in the same services where they are also users.

2. Training MHPs to work with PSWs

According to the data, the training of MHPs is not only a measure to safeguard PSWs but also a prerequisite for the effective implementation of PSWs. Another pivotal element that emerged is that training, to be effective, must be conducted by or with individuals with lived experience. Moreover, training should have a purpose that extends beyond mere knowledge of peer support and PSWs’ activity profile and should inspire a change in perspective, leading to the overcoming of stigma and prejudice.

3. Institutional recognition of PSWs

To effectively and meaningfully implement peer support, institutional recognition of the professional figure by the Ministry of Enterprises and Made in Italy is essential. This would clarify the role of PSWs and enable proper integration of PSWs into services through appropriate contracts, clear job expectations and sufficient organisational support. In 2024, a professional association (AIPESP^2^) was established in Italy to promote the institutional recognition of the PSW role. According to the respondents, such recognition could help safeguard their labour rights, create dedicated job opportunities, and, most importantly, affirm the legitimacy and societal value of their work.

4. Synergy between motivation at the operational level and institutional support

Many of the interviewees believe that there is a need for greater synergy between motivation at the “operational” level (on the part of PSWs and professionals) and interest at the “strategic” level. The inclusion of PSWs at the strategic level implies the development of co-produced pathways in which PSWs are called upon, alongside professionals, to build services that meet users’ needs and expectations. For example, according to the new regional directives, mental health services in Friuli Venezia Giulia will be required to collaborate primarily with social cooperatives that employ PSWs as part of their staff.

5. Enhancing experiential knowledge in decision-making spaces and research context

The interviewees emphasised the need to recognise experiential knowledge as equally important as technical-academic expertise, arguing that its integration could enhance clinical practices by making them more responsive to individuals’ lived experiences.

*“The experience of these individuals should be made comparable to that of the professional. What you read in a book and what you experience firsthand could be two parallel systems of knowledge”. (MHP4).*

6. Cultural and epistemological change in mental health services

The key issue that emerged from the interviews and participant observation is that the inclusion of PSWs in mental health services requires a profound cultural shift. This change involves rediscovering a genuine commitment to supporting individuals and their lived experiences and, according to some, recognising that many forms of suffering in mental health are primarily social and cannot be addressed only through a narrow biomedical approach.

*“If you deal with mental health*,* you cannot but deal with social justice. Let’s talk about social justice! We are talking about abuse*,* violence*,* discrimination*,* marginality*,* and abandonment. […] That is*,* you cannot but reflect on the fact 'What can I do to give these people a perspective?'”. (MHP8).*

## Discussion

### Trieste as an Advanced Context for Critical Reflection

Compared to other studies based on either qualitative designs or outcome evaluations, our research offers a distinct contribution by examining these tensions in a historically advanced and recovery-oriented system. As suggested by Gillard et al. (2024), it is essential to move beyond assessing the effects of peer support and instead investigate the conditions under which peer roles become meaningful and effective. In this regard, studying the case of Trieste—a setting characterised by a *no-restraint*, *open-door* approach and a strong cultural commitment to deinstitutionalisation—offers a unique vantage point (Mezzina, 2014, 2016). The relative absence of structural barriers typically found in more medicalised or hierarchical systems enables a clearer focus on the epistemic and relational tensions that persist between professional and experiential knowledge. Rather than masking contradictions, this context allows them to emerge more visibly, making it a privileged site for critical reflection on the institutional inclusion of peer support. Consequently, this study contributes to the international conversation by offering context-sensitive insights that are both grounded and transferable, especially for services seeking to move toward more participatory and person-centred approaches.

### The Value of Peer Support in Community care

The profile of PSWs in the community-based mental health services of Friuli Venezia Giulia appears to be broadly consistent with the values and practices described in international literature. The findings suggest that PSWs contribute to several aspects of personal recovery, including increased self-efficacy and self-esteem (Cooper et al., 2024) and empowerment (Burke et al., 2019; Peck et al., 2023), reducing self-stigma (Burke et al., 2019) and loneliness (Thomas et al., 2023), and fostering feelings of hope and enhanced quality of life (Cooper et al., 2024; Davidson et al., 2012; Lloyd-Evans et al., 2014; Pitt et al., 2013; Repper & Carter, 2011a).

Compared to other contexts, in Trieste PSWs enhance communication between service users and professionals, facilitating mutual understanding and shared decision-making, particularly in situations where users face difficulties in expressing concerns or questioning clinical recommendations (Lennox et al., 2021; Ruiz-Pérez et al., 2025). Moreover, in this region, the role of PSWs as mediators is not limited to service settings; rather, their contribution extends to acting as intermediaries between mental health services and the broader community. Indeed, in this context, PSWs adopt a clear community-oriented approach: their focus on group work and social reconnection indicates that their contribution extends beyond individual recovery to include community empowerment, driven by a strong commitment to advocating for the rights and participation of people with mental disorders (Burke et al., 2019; Høgh Egmose et al., 2023). Consequently, PSWs emerge as key players in the social inclusion of people with complex needs, facilitating the construction or restructuring of a social network and acting as intermediaries between the person and their community (Bologna & Simmons, 2018; Davidson et al., 2012; White et al., 2020).

In Friuli Venezia Giulia, PSWs also contribute to contrast stigmatising narratives around mental illness. Through their advocacy and awareness-raising, they challenge stigma both within services and in broader society, offering a living example of recovery and active citizenship (Burke et al., 2019). This redefinition of identity and value supports what anthropologists refer to as “symbolic efficacy,” where healing involves making meaning of suffering (Pizza, 2005)^3^.

Furthermore, in line with previous literature (Repper, 2013; World Health Organisation, 2019), in this context it is particularly evident how PSWs impact the implementation of quality mental health care practices by promoting care humanisation, person-centred and rights-based approaches; improving user–professional dialogue, and inviting services to adopt a more participatory stance (Burke et al., 2019; Cleary et al., 2018; World Health Organisation, 2019). Finally, our study highlighted the positive impact of peer support on PSWs’ own recovery (Poremski et al., 2022; Repper, 2013).

### Challenges for Implementation

Despite these promising aspects, our study also revealed a number of critical challenges, echoing concerns in the wider literature (Davidson et al., 2012; Kemp & Henderson, 2012; Repper & Carter, 2011b). Among these, stigma and prejudice remain significant, often expressed through subtle forms of professional resistance. This may include protective attitudes, or a tendency to not recognise the contribution of PSWs (Cooper et al., 2024). Furthermore, the study highlights the subtle risks of using PSWs to improve user compliance or enhance the public image of services, without fully integrating their voices or perspectives. These dynamics suggest ongoing challenges related to power-sharing, recognition, and the legitimisation of experiential knowledge (Slade et al., 2014; Thomas et al., 2023). A key challenge to fostering meaningful inclusion lies in the persistence of hierarchical structures and the limited exploration of power redistribution within mental health systems. Even within advanced mental health systems, such as that of Friuli Venezia Giulia, peer support may be at risk of being implemented in a tokenistic or low-cost manner, consequently losing its potential for critical and transformative impact (Ruiz-Pérez et al., 2025). In these contexts, peer support can still offer valuable contributions, but risks shifting from a relational and transformative practice to a more technical role, with its emancipatory potential significantly constrained by the structural conditions in which it is implemented (Bologna & Simmons, 2018; Rose, 2014; Rose & Rose, 2023; Thomas et al., 2023).

Consequently, our study emphasises the importance of supporting PSWs within mental health services. To address these issues, the study identifies several concrete strategies: establishing clear roles, ensuring supportive supervision, promoting collaborative practices, and providing adequate training for both PSWs and professionals (Brown et al., 2024; Burr et al., 2020; Cooper et al., 2024; Davidson et al., 2012; Kemp & Henderson, 2012; White et al., 2020). In this regard, according to our findings, one structural safeguard against the risk of co-optation has been identified in the establishment of institutional spaces for ongoing dialogue with communities—such as service user and family participation committees—where PSWs can engage with diverse and, at times, critical perspectives (Curwen et al., 2019). These settings may help maintain a reflective stance on service practices and support the development of a more independent and community-responsive peer role. Moreover, another key issue is the institutional recognition of PSW role in order to guarantee employment rights and formally validate experiential knowledge as a legitimate and necessary component of care (Reeves et al., 2024). While we consider this process crucial, in line with other studies, we also acknowledge the risk that professionalisation may undermine the peer nature of relationships and lead to the institutional assimilation of PSWs (Burke et al., 2019; Gillard et al., 2024). Institutional recognition, however, is not merely a bureaucratic necessity; it must be accompanied by strong managerial commitment and adequate resource allocation to foster an environment conducive to effective collaboration.

Without these conditions, the inclusion of PSWs risks being superficial, reinforcing conventional service models rather than fostering genuine peer support-oriented programmes. In this sense, assuming that PSW integration alone can radically transform traditional services—particularly those not oriented toward a person-centred and community-based approach (Gillard et al., 2024)—may inadvertently lead to the development of standardised programmes delivered by PSWs rather than initiatives truly grounded in peer support principles (Bologna & Simmons, 2018; Gillard et al., 2017).

### Implementation Perspectives for Research and Service Organisation

According to our results, a valuable strategy for an effective implementation of PSWs should consist of involving them not only in daily rehabilitation practices but also in service design, research and evaluation. Their insights can guide innovation across organisational levels and contribute to more responsive, inclusive care models (Thomas et al., 2023; Zisman-Ilani & Byrne, 2023). This would imply organisational commitment that can guide resource allocation, service design, evaluation and management *for* and *with* PSWs (Reeves et al., 2024). On the other hand, it is important that PSWs are enabled to develop and express critical viewpoints regarding the full spectrum of service decisions, including those typically associated with clinical authority (Ruiz-Pérez & von Peter, 2025). Limiting their involvement to psychosocial or rehabilitative activities may unintentionally reinforce existing professional boundaries and leave unquestioned the assumptions underpinning core areas of care. Creating space for PSWs to participate in reflective dialogues around all aspects of service provision can help ensure that peer support does not become a marginal or tokenistic element within established clinical frameworks, but instead contributes to reshaping the culture of care in more inclusive and dialogical ways.

For instance, in Friuli Venezia Giulia, the implementation of training initiatives and interdisciplinary working groups involving professionals and managers has been proposed, with the aim of fostering the critical and meaningful inclusion of the experiential perspective of PSWs—and, more broadly, of service users and family members—within mental health services. Such efforts should, however, be accompanied by targeted training for mental health professionals, aimed at raising awareness of existing hierarchies and power asymmetries within care settings.

Moreover, we argue that while the work of PSWs can encourage professionals to reflect on and humanise their practices, cultural change is a necessary condition for PSWs to perform their roles effectively (Brown et al., 2024; Cooper et al., 2024; Gillard et al., 2022). Cultural change requires an acknowledgement that this complexity of the experience of suffering can only surface through listening to and valuing lived experiences (Rose & Rose, 2023). The effective engagement of PSWs can be a cornerstone of this transformation, offering an opportunity to catalyse these reflections, as it provides a chance to shift the lens through which mental health suffering is understood. In summary, paraphrasing Diana and Nikolas Rose, this shift must transition from “involving individuals in services” to “involving services in the lived world of individuals” (Rose & Rose, 2023).

### Limitations

This study is characterised by an ethnographic design, which inherently prioritises depth over breadth. As such, its findings are not intended to be generalisable in a statistical sense. Rather, the aim is to produce a situated and critical understanding of practices and their implications within a specific context. While the uniqueness of the setting limits the replicability of the study, we believe that the insights generated may nevertheless offer valuable reflections for services operating in similar socio-cultural and organisational environments, such as community mental health services.

At the same time, the favourable orientation of the Trieste model toward recovery, deinstitutionalisation, and non-coercive care may have shaped the implementation of peer support in ways that are not easily transferable to more hierarchical or medicalised systems. In such settings, some of the challenges observed here—such as role ambiguity, professional resistance, or the marginalisation of experiential knowledge—might be even more pronounced. Conversely, we also found that the *symbolic capital* of advanced models may obscure residual power asymmetries, making them more difficult to challenge. For this reason, we believe that the study’s findings may be relevant not only for systems in earlier stages of peer support integration, but also for those that consider themselves already aligned with recovery-oriented values.

A further limitation concerns the composition of the research team. Although the team has long-standing experience in participatory research and in working alongside individuals with lived experience, none of the researchers involved in the design and data collection processes identify as a Peer Support Worker. To mitigate this limitation, findings were discussed with participants—including PSWs—during the analysis phase. Nevertheless, we acknowledge that the absence of PSWs in key stages of the research process represents a significant constraint, particularly in a study aiming to explore their roles and experiences.

While this study recognises the potential of peer support to promote institutional critical reflexivity and service transformation, it also acknowledges that this potential depends on the specific organisational, cultural and relational context in which peer support is embedded. The implementation of PSWs is not inherently transformative; rather, the effects depend on services’ willingness and ability to redistribute power, legitimise experiential knowledge and engage in continuous critical reflexivity. Further comparative research is necessary to examine how different service cultures and structures shape the role, recognition and impact of PSWs in various contexts.

## Conclusions

This ethnographic study contributes to the growing body of research on peer support by examining its implementation within a mental health system historically shaped by the principles of deinstitutionalisation and community-based care. Through an in-depth analysis of the Friuli Venezia Giulia region, it identifies both enabling factors and structural challenges that influence the meaningful inclusion of PSWs. In contrast to much of the existing literature, this research sheds light on the epistemic tensions and risks of co-optation that can emerge even within progressive and participatory service models. By integrating the perspectives of both PSWs and mental health professionals, the study illustrates how peer support can serve as a catalyst for institutional self-reflection, while also showing that its transformative potential depends on broader cultural and organisational change.

## Data Availability

Data availability: the qualitative data (interview transcripts and field notes) generated and analysed during the current study are not publicly available due to concerns regarding participant confidentiality and privacy. However, anonymised data extracts may be made available from the corresponding author upon reasonable request. Materials and code availability: while no specific software or computational code was developed for this study, the coding framework and thematic structure generated through the framework analysis are available from the corresponding author upon reasonable request.

## Footnotes

1 AIPESP is currently engaged in discussions with the Ministry concerning the formal recognition of the PSW role, for which the association is responsible for securing ministerial approval. This initiative is part of a broader range of activities undertaken by AIPESP. For further information, please visit: www.aipesp.it.

2 AIPESP is the acronimous of “Associazione Italiana Persone Esperte in Supporto tra Pari” (trad. Italian association of PSWs). For fourther information please consult https://aipesp.it/.

3 According to this concept, healing involves the construction of a *representational space* that gives meaning to one’s experience of illness (Pizza, 2005).
